# Nordic textile anatomy database: Composition of garments available in the nordic retail mass market and post-consumer textile waste market

**DOI:** 10.1016/j.dib.2025.111512

**Published:** 2025-03-23

**Authors:** Heather Margaret Logan, Maggie Ziggie Søndergaard, Valentina Rossi, Kamilla Kastrup Hansen, Anders Damgaard

**Affiliations:** Technical University of Denmark, Bygningstorvet, Bygning 115, Kgs, Lyngby 2800, Denmark

**Keywords:** Disruptors, Consumer goods, Waste composition, Recycling, Industrial ecology, Waste management

## Abstract

Textiles are complex materials made of multiple and blended resources, often assembled in unique configurations imbuing each textile with its own anatomy. These ***textile anatomies*** make the identification, separation, sorting, and recycling of post-consumer textiles, especially, difficult. While textile anatomy data is often retrievable for pre- and post-industrial textiles (off-cuts or rejects from manufacturing), it is often difficult to retrieve for textiles which have reached the consumer. The lack of data available on the textile anatomies of pre- and post-consumer textile waste skews predictions and market forecasts for the expected yields, capacities, and qualities of post-consumer textile sorting and recycling activities. This disrupts planning for sorting, removal of findings, and scaling of recycling technologies. To better plan for the innovation needs, market capacity, and policy levers needed to improve the efficiency of sorting and recycling activities, there is an urgent need for data on the unique anatomies of pre- and post-consumer textiles. This is especially important as the EU mandates that all member states must separately collect and treat post-consumer textiles beginning in 2025.

Therefore, this database contains two datasets offering textile anatomies for more than 5000 separate garment samples from the post-industrial-pre consumer retail mass market (RMM) and the Post-Consumer Textile Waste Market (PCTWM). This database contains crucial data on each garment's fibre composition, finding presence, and layer presence. The two datasets are the results of two separate waste composition campaigns conducted in the Nordic Region in 2022: One focused on the textile anatomies of the RMM (4,495 samples) and the other on the PCTWM (1,248 samples). The RMM data was collected by sampling garments across mass market retailers in the Copenhagen municipality of DK during the spring/summer seasons of 2022. The PCTWM data was collected by sampling post-consumer textile waste bales from pre- and post-sorting lines at the SIPTEX sorting facility in Malmo, SE in the winter of 2022. In both datasets, surveys deployed via webapp were utilized to streamline sampling and ensure consistent recording of the fibre blends, number of findings present, and layers present. In the PCTWM dataset additional data is provided on the fibre composition of layers, as well as the placement and type of findings present.

Each dataset in this database can be used by industrial ecologists, economists, and textile engineers to better forecast, map, and analyse the potential treatment of expected post-consumer textiles. Moreover, the methodology and approach to data gathering can be used as a blueprint for future regionalized databases throughout the European Union. The use of this database can be particularly useful to analyse the economic, environmental, and resource impacts of common garments as well as inform textile market analysis, design guidelines, and policy decisions for the treatment of post-consumer textile waste in the circular economy.

Specifications table

**Table:** 

Subject	Environmental Science
Specific subject area	Textile Waste Composition and Management
Data format	**Filtered .csv file (dataset with labels)**
Type of data	Table
Data collection	The database contains 2 datasets each of which was collected via web-deployed surveys designed to aid direct sampling and composition analysis of textiles: (1) At retail stores in the RMM in DK. a. At each location, 10 textiles across 10 categories were sampled. Fibre blend, lining presence, and number of findings were recorded. Data was cleaned to ensure grammatical correctness, fibre order, and removal of incomplete survey entries. (2) Of the PCTWM at the SIPTEX plant in SE. a. 2 bales of pre- and 2 bales of post-sorted textiles were sampled. Fibre composition, lining composition, and placement/number of findings were recorded. Data was cleaned as in dataset 1.
Data source location	Dataset RMM was collected in Copenhagen DK, and dataset PCTWM was collected in Malmo, SE. The final database is stored at the Technical University of Denmark's open-source data repository, DTU Data.
Data accessibility	Repository name: DTU Data Data identification number: 10.11583/DTU.24581700 Direct URL to data: 10.11583/DTU.24581700.v1
Related research article	

## 1 Value of the Data

• Provides a comprehensive overview of the fibre blends common in textiles, particularly garments, by percentage per fibre.
• Provides a detailed overview of the presence of layers and recycling disruptors in garments entering the retail mass market (RMM) and Post-Consumer Textile Waste Market (PCTWM).
• Provides details on the placement and type of disruptors present in the PCTWM, useful for planning sorting, cutting, shredding, and possible recycling yields.
• Provides data for generating representative inventories for Life Cycle Assessments, Material Flow Analyses, or other such models for assessing the flows of resources and the environmental impacts associated with textiles.

## 2 Background

To date, most studies producing data on textiles focus on garment composition from the perspective of single fibres alone [[1], [2], [3], [4], [5]], meaning often only the leading (or majority share) fibre is recorded. Many of these studies also omit or aggregate observations on linings and disruptors [[2], [3],^5^]. This limits planning for recycling and sorting needs as, textiles are most often composed of blended fibres and contain layers and findings (trims, zippers, buttons, etc.). ***Textile anatomies*** play a vital role in assessing the treatment needed, quality, and substitution potential of textiles in the circular economy [5]. Thus, this database fills a significant knowledge gap in the field of industrial ecology on the anatomical composition of textiles [6,7] for the RMM and PCWTM by providing data on individual textiles which incorporate their fibre blends, layers, and findings.

The textiles were sampled in the RMM and PCWTM to avoid bias in the composition analysis from cherry-picking from the company-provided samples, planned behavior bias from consumer self-reporting, and selection bias from wardrobe studies. This database contains textile anatomy data including labeling, fibre blends by percentage, findings, and lining attributes of common textiles. This data improves the granularity of inventories and aids the mapping of resources in post-consumer textile futures [8].

## 3 Data Description

This database provides a representative sampling of garments in RMM and PCTWM as two separate datasets. Each dataset details the garment type following Common Nomenclature guidelines [9], the fiber composition, and the presence of linings and disruptors for each sample. In addition, the PCTWM dataset includes the composition of linings, disruptor type, and disruptor placement. All metrics are described in Table 1 for the RMM dataset and Table 2 for the PCTWM dataset. The final datasets were cleaned to ensure standardization in the spelling of fiber names using EU labeling guidelines [10] and to remove incomplete samples due to input errors during the field sampling.

**Table 1:** Column headings and description for retail mass market (RMM) dataset.

Column	Description
Textile Category	This column denotes the textile category of each sample. The textile categories are defined in Table 3 and adhere to EU CN Definitions for Imports and Exports of Textiles [9].

**The following Columns occur in groups of 3, each with the following headings per fibre [Count]. There were at most 5 Fibre types found in this survey, therefore the [COUNT] occurs between numbers 1-5.**	

**Fiber ID [COUNT]**	Fibre ID is based on the name declared on the label and adheres to the numbering system utilized in the EU fibre label declaration guidelines [10]. See Table 4 for a full list.
**Fiber Name [COUNT]**	Fibre type is reported from the labels available on the sampled garments and adhere to the naming system utilized in the EU fibre label declaration guidelines [10]. See Table 4 for a full list.
**Fiber % [COUNT]**	Percent each fibre contributes to the total garment composition as detailed on the fibre declaration and care label.

**The following columns occur as normal.**	

**Total Findings**	Numeric. The number of findings such as zippers, rivets, buttons, trims, and embellishments present on the garment is denoted in this column.
**Layer Presence**	Boolean. The presence of a lining or additional layers is denoted in this column.
**Survey**	This column details if the sampling was conducted during the first or second period.

**Table 2:** Column headings and description for Post-Consumer Textile Waste Market (PCTWM) Dataset.

Column	Description
**Item ID**	This column denotes the ID of the sampled item.
**Sorting Status**	This column denotes if the sample was taken from the pre- or post-sorted bales.
**Bale Composition Label**	This column denotes the intended composition of the bale. Pre-sorted bales were “*100 % Mixed”*, while post-sorted bales were either intended to be “*90 % cotton”* (meaning 90 *%* or greater) or “*60 % polyester”* (meaning 60 *%* or greater).
**Article Color**	Each sample can only be one of the following colors: *white, yellow, orange, red, purple, blue, green, grey, brown, black, multi-color pattern,* or *multi-color striped*.
**Article Category – EU CN**	EU Common Nomenclature definition for product group [9]. See Table 3 for the full list. “*Othe*r” denotes items not included in Table 3.
**Article Category General**	The general category grouping for each sample: *accessories, adult, babies, children,* or *other*.
**Article Category Detail**	Details on the specific sample type. May be any of the following: *Hats and Headwear; Small Accessories; Medium Accessories; Denim Trousers; Trousers (woven); Shorts, Bermuda Shorts; Denim Shorts; Denim Dresses, Overalls, and Jumpsuits; Dresses; Shirts, Blouses (Woven); Lightweight Jackets; Sweaters (Knits); T-Shirts and Polos (knits); Denim Skirt; Bras and Lingerie; Shorts, Bermuda Shorts; Household linen; Skirts; Sports Trousers (knits); Homewear; Denim jackets; All bottoms; Socks and Hosiery; Clothes; Underwear and Accessories; Other;* or *Fabrics by the Meter*
**Fibre Label Presence**	Denotes the presence and status of the label: *Yes, a tag is present; No, there is no evidence of a fibre label; No, the tag has been removed; No, the printed label is worn; Yes, but the tag printing is too worn to read;* or *Yes, a printed label.*

**The following Columns occur in groups of 3, each with the following headings per fibre [Count]. There were at most 5 Fibre types found in this survey, therefore the [COUNT] occurs between numbers 1-5.**	

**Fiber ID [COUNT]**	Fibre ID is based on the name declared on the label and adheres to the numbering system utilized in the EU fibre label declaration guidelines [10]. See Table 4 for a full list.
**Fiber Name [COUNT]**	Fibre type is reported from the labels available on the sampled garments and adhere to the naming system utilized in the EU fibre label declaration guidelines [10]. See Table 4 for a full list.
**Fiber % [COUNT]**	Percent each fibre contributes to the total garment composition as detailed on the fibre declaration and care label. This column denotes the fibre composition as it was written on the label; if no label is present or the label is unreadable, this column is marked as “*No Fibre Data.”*

**The following columns occur as normal.**	

**Layers**	Articles can be Mono or Multi-layered
**laYER Included in Label?**	Indicates if the fibre content of the lining is declared on the label. This column may include: *Yes; No;* or *No layer.*
**Findings and/or trims?**	Boolean. Denotes if there are findings or trims present.

**The following 4 Columns denote the same data per Finding Type**	

**Total [Finding Type] Present**	Numeric. These columns denote the total number of finding types present on the article/garment. Finding types include metal, plastic, fabric, and other materials.

**The following columns occur in groups of 17. This is LED by the detailed finding type and followed by the placement of that finding type.**	

***[Finding Type]*_[Detail]**	These columns denote the finding general group: *metal, plastic, fabric,* or *other,* followed by an “_” and the detailed finding type: *buckle, button, charm, eyelets, fastener, pearls wire, hook and eyelet/bar, rivet, snap buttons, zipper, designer's label, elastic, embroidery, patch, exterior pocket (alternative fabric), inserts and adornments, string, leather, prints,* or *misc.* The use of *“N/A”* indicates that the particular finding group and type is not present on the article/garment.
**[Finding Type]_[Detail]_[Placement]**	Boolean. Denotes where the [FINDING TYPE]_[DETAIL] is located in the article/garment. Articles/garments are split into four quadrants on both the front and four quadrants on the back, starting with *Q1* in the upper left-hand side facing the surveyor and continuing clockwise. In addition, *vertical fold*s, *horizontal fold*s, *sleeves, collar*s, and *hem*s are considered their own areas. An illustration of this system is provided in Fig. 3.

The final RMM dataset includes data for 4,495 sampled garments across ten textile categories. The data set is available as a .csv file from the linked repository. The final database is stored in the textileanatomy_database_RMM.csv file [11].

The final PCTWM dataset includes data for 1248 sampled garments, building upon the textile categorization method developed for the RMM dataset. The PCTWM dataset has slightly higher granularity than the RMM dataset as it includes details on the label status, finding types, and placement of findings for each sample. The dataset is available as a .csv file from the linked repository. The final database is stored in the textileanatomy_database_PCTWM.csv file [11].

This data is intended to be used to construct representations of textiles considering trends in textile anatomy per the common textile categories found in the EU. While this database is the most representative of the Nordic region, it provides valuable data needed to model fibre blends, find presence, lining presence, and even finding placement for many textile categories which are crucial in creating models of textile flows as exemplified in [8]. These models could also be extrapolated to better represent the import and export statistics per textile category or fibre type most representative to other geographical regions, such as other EU member states. Such extrapolations can then be used to model exploratory MFAs and LCAs of prospective futures for textile collection and treatment in line with EU initiatives and policy planning. While not fully representative of all other regions, such models can serve as baseline processes allowing decision makers to hotspot where further region-specific data could be useful in improving collection guidelines or technological development for their geographic regions. Approaches like this are commonly used to create LCA datasets for databases.

## 4 Experimental Design, Materials and Methods

This database results from two extensive field studies of garments in the DK and SE textile markets with a focus on ready-made garments for mass consumption. In both the RMM and PCTWM datasets, textiles may originate from mass-market, mid-level, and high-end high-street retailers who sell ready-made garments. The RMM dataset excludes garments from value, diffusion, luxury, haute-couture, and hand-made retailers. It is assumed that the two datasets contain similar types of garments, but this cannot be confirmed in the PCTWM dataset due to the number of damaged, worn, or removed fibre and brand labels from the samples. In the RMM dataset, this scoping was chosen to ensure the best representation of textiles in the “average” DK household and avoid textiles that may be manufactured with alternative or longevity approaches which are not necessarily representative of the textile anatomies of mass-market garments. It is important to note that this database only offers data on the anatomy of these garments, providing only physical characteristics, and does not include emotional or durability indicators.

In both datasets, physical characteristics are recorded from the label accompanying the garments, whether the label was attached physically to the garment and/or printed on the garment in accordance with the EU Regulation 1107/2011 [10]. The garments were sampled in the field and recorded via mobile-phones and computers using web-deployed surveys. Only incomplete survey responses were removed from the final datasets. The data has then been standardized between the two datasets to ensure consistent spelling, nomenclature, and order of reporting across the database.

### 4.1 RMM Sampling

In the RMM dataset samples were selected from retail locations in Copenhagen, DK, which adhered to the mass market description provided above. In addition, each location was determined to have two or more locations within the municipality. This was done to ensure the selected garments would be representative of the average consumer and not representative of a single shopping environment (i.e. high end, novelty, outlet, indie, etc.).

In each location, the field teams selected ten items each from each of the ten primary garment categories imported yearly to DK [3]. These categories and their definitions are listed and described in Table 3 and are adopted from the combined nomenclature (CN) EU import codes and definitions [9]. Items were chosen using the category definitions and each of the samples was selected from various locations throughout the store. This approach to sample selection helped ensure garments in each category were selected from a variety of collections available in the store. In some locations less than ten categories could be sampled as some retailers only cater to a selected audience. In this case, the unavailable categories were omitted from the sampling for this location.

**Table 3:** Textile garment categories included in Survey. This table denotes the textile categories considered, their definition, and the import code for the EU and is modified from [11].

Product group	4-digit CN Codes	2-Digit Product Code	Category Definition [9] (Annex I: pp. 378-397)
**Dresses and skirts**	6104	41, 42, 43, 44, 49, 51, 52, 53, 59	"Women's or girls' dresses, skirts, divided skirts, knitted or crocheted"
	6204	41, 42, 43, 44, 49, 51, 52, 53, 59	"Women's or girls' dresses, skirts, divided skirts"
**Handkerchiefs, ties, scarves, gloves, and other**	6116	10, 91, 92, 93, 99	"Gloves, mittens and mitts, knitted or crocheted"
	6117	10, 80, 90	"Other made up clothing accessories, knitted or crocheted; knitted or crocheted parts of garments or of clothing accessories"
	6213	20, 90	"Handkerchiefs"
	6214	10, 20, 30, 40, 90	"Shawls, scarves, mufflers, mantillas, veils and the like"
	6215	10, 20, 90	"Ties, bow ties and cravats"
	6216	00	"Gloves, mittens and mitts"
**Overcoats and anoraks**	6101	20,30,90	"Men's or boys' overcoats, car-coats, capes, cloaks, anoraks (including ski-jackets), windcheaters, wind-jackets and similar articles, knitted or crocheted, other than those of heading No 6103"
	6102	10, 20,30,90	"Women's or girls' overcoats, car-coats, capes, cloaks, anoraks (including ski-jackets), windcheaters, wind-jackets and similar articles, knitted or crocheted, other than those of heading No 6104"
	6201	20,30,40,90	"Men's or boys' overcoats, car-coats, capes, cloaks, anoraks (including ski-jackets), windcheaters, wind-jackets and similar articles, other than those of heading No 6203"
	6202	20, 30, 40, 90	"Women's or girls' overcoats, car-coats, capes, cloaks, anoraks (including ski-jackets), windcheaters, wind-jackets and similar articles, other than those of heading No 6204"
**Shirts, blouses, tops**	6105	10, 20, 90	"Men's or boys' shirts, knitted or crocheted"
	6106	10, 20, 90	"Women's or girls' blouses, shirts and shirt-blouses, knitted or crocheted"
	6205	20, 30, 90	"Men's or boys' shirts"
	6206	10, 20, 30, 40, 90	"Women's or girls' blouses, shirts and shirt-blouses"
**Sportswear and swimwear**	6112	11, 12, 19, 20, 31, 39, 41, 49	"Track suits, ski suits and swimwear, knitted or crocheted"
	6211	11, 12, 20	"Track suits, ski suits and swimwear; other garments"
**Suits and blazers**	6103	10, 22, 23, 29, 31, 32, 33, 39	"Men's or boys' suits, ensembles jackets, blazers, knitted or crocheted"
	6104	13, 19, 22, 23, 29, 31, 32, 33, 39	"Women's or girls' suits, ensembles, jackets, blazers, knitted or crocheted"
	6203	11, 12,19, 22, 23, 29, 31, 32, 33, 39	"Men's or boys' suits, ensembles jackets, blazers"
	6204	11, 12,13, 19, 21, 22, 23, 29, 31, 32, 33, 39	"Women's or girls' suits, ensembles, jackets, blazers"
**Sweaters and cardigans**	6110	11, 12, 19, 20, 30, 90	"Jerseys, pullovers, cardigans, waistcoats and similar articles, knitted or crocheted"
**Trousers and shorts**	6103	41, 42, 43, 49	"Men's or boys' trousers, bib and brace overalls, breeches and shorts (other than swimwear), knitted or crocheted"
	6104	61, 62, 63, 69	"Women's or girls' trousers, bib and brace overalls, breeches and shorts (other than swimwear), knitted or crocheted"
	6203	41, 42, 43, 49	"Men's or boys' trousers, bib and brace overalls, breeches and shorts (other than swimwear)"
	6204	61, 62, 63, 69	"Women's or girls' trousers, bib and brace overalls, breeches and shorts (other than swimwear)"
**T-shirts, singlets and vests, hoodies and crewnecks**	6109	10, 90	"T-shirts, singlets and other vests, knitted or crocheted"
**Underwear, socks, night clothes**	6107	11, 12, 19, 21, 22, 29, 91, 99	"Men's or boys' underpants, briefs, nightshirts, pyjamas, bathrobes, dressing gowns and similar articles, knitted or crocheted"
	6108	11, 19, 21, 22, 29, 31, 32, 39, 91, 92, 99	"Women's or girls' slips, petticoats, briefs, panties, nightdresses, pyjamas, négligés, bathrobes, dressing gowns and similar articles, knitted or crocheted"
	6115	10, 21, 22, 29, 30, 94, 95, 96, 99	"Panty hose, tights, stockings, socks and other hosiery, including stockings for varicose veins and footwear without applied soles, knitted or crocheted"
	6207	11,19, 21, 22, 29, 91, 99	"Men's or boys' singlets and other vests, underpants, briefs, nightshirts, pyjamas, bathrobes, dressing gowns and similar articles"
	6208	11, 19, 21, 22, 29, 91, 92, 99	"Women's or girls' singlets and other vests, slips, petticoats, briefs, panties, nightdresses, pyjamas, negligés, bathrobes, dressing gowns and similar articles"
	6212	10, 20, 30, 90	"Brassières, girdles, corsets, braces, suspenders, garters and similar articles and parts thereof, whether or not knitted or crocheted"

**Fig. 1:**
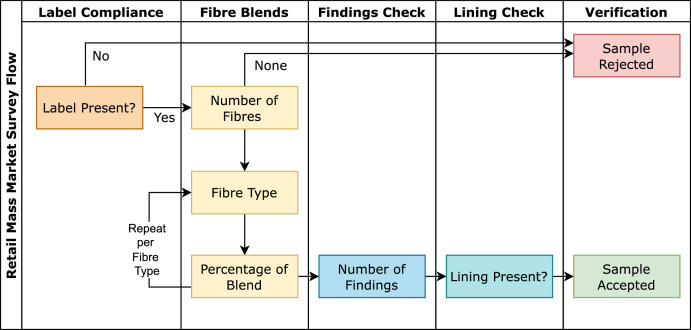
Flow of survey questions to aid in retail mass market (RMM) field sampling. These questions include a disclosure of the textile category, the presence of a finding declaration and care label, the fibre composition, lining presence, and finding presence.

Each garment was then assessed by hand using a systematic method which was aided by a digital survey for consistency. The sampling method for the survey adheres to the hierarchy illustrated in Fig. 1 and included six primary criteria: *Textile Category, Fibre Declaration and Care Label Presence, Fibre Types, Fibre Composition, Finding Presence*, and *Lining Presence*.

The teams first checked for the presence of a fibre declaration and care label for each garment. Fibres were noted from the care label and recorded by selecting the applicable fibre list from the EU Fibre Declaration Regulation [10]. A List of these fibres is available in Table 4. The researchers then input the relative percentage of each fibre to yield a comprehensive overview of the overall fiber composition of each garment. Garments which did not have a fibre declaration and care label or did not list any of the allowed fibre types on the label were excluded from sampling.

**Table 4:** Fibre types and identification number. This table is modified from EU 1107/2011 regulation [10].

Fibre ID	Fibre Type	EU 1107/2011 Approved Name [10]
**1**	protein	Wool
**2**	protein	alpaca, llama, camel, cashmere, mohair, angora, vicuna, yak, guanaco, cashgora, beaver, otter, followed or not by the word ‘wool’ or ‘hair’
**3**	protein	animal or horsehair, with or without an indication of the kind of animal
**4**	protein	Silk
**5**	cellulose	Cotton
**6**	cellulose	Kapok
**7**	cellulose	flax (or linen)
**8**	cellulose	true hemp
**9**	cellulose	Jute
**10**	cellulose	abaca (Manila hemp)
**11**	cellulose	Alfa
**12**	cellulose	coir (coconut)
**13**	cellulose	Broom
**14**	cellulose	Ramie
**15**	cellulose	Sisal
**16**	cellulose	Sunn
**17**	cellulose	Henequen
**18**	cellulose	Maguey
**19**	man-made cellulose	Acetate
**20**	man-made cellulose	Alginate
**21**	man-made cellulose	Cupro
**22**	man-made cellulose	Modal
**23**	man-made protein	Protein
**24**	man-made cellulose	Triacetate
**25**	man-made cellulose	Viscose
**26**	man-made synthetic	Acrylic
**27**	man-made synthetic	Chlorofibre
**28**	man-made synthetic	Fluorofibre
**29**	man-made synthetic	Modacrylic
**30**	man-made synthetic	polyamide or nylon
**31**	man-made synthetic	Aramid
**32**	man-made synthetic	Polyimide
**33**	man-made cellulose	Lyocell
**34**	man-made synthetic	Polylactide
**35**	man-made synthetic	Polyester
**36**	man-made synthetic	Polyethylene
**37**	man-made synthetic	Polypropylene
**38**	man-made synthetic	Polycarbamide
**39**	man-made synthetic	Polyurethane9
**40**	man-made synthetic	Vinylal
**41**	man-made synthetic	Trivinyl
**42**	man-made synthetic	Elastodiene
**43**	man-made synthetic	Elastane
**44**	other	glass fibre
**45**	man-made synthetic	Elastomultiester
**46**	man-made synthetic	Elastolefin
**47**	other	Melamine
**48**	other	material of which the fibres are composed, followed or not by the word ‘yarn’ or ‘fibre’
**49**	man-made synthetic	polypropylene/polyamide bicomponent
**50**	man-made synthetic	Polyacrylate

Following the fibre composition and label identification, each garment was visually assessed for the presence of linings and findings. Linings are fabric-based interfacing or layers used to stabilize the garment or improve the fit, performance, or thermal comfort of the garment [5,12]. Linings are often made of different fibre types than the main garment [12] and are a barrier to recycling as they are difficult to remove which may lead to contamination of recycling and ultimately reduce recycling efficiency and the quality of the recyclate [13]. Moreover, due to their placement on interior layer of clothing, they cannot be easily detected with automated sorting machines [5] this may lead to reduction in the value of sorted bales as they need additional sorting and separation before recycling. Findings are zippers, buttons, hooks and eyes, trims, and other embellishments used to aid closure, improve functionality, or add to the aesthetic of the garment [5]. Findings can disrupt recycling by damaging machinery, and therefore often need to be removed or may disqualify the entire garment from recycling [5,13]. When not accounted for in data on the flows of textiles to recycling routes, the total mass of materials available for recycling can be underestimated. Data on the fibre blend, lining presence, and finding type is critical to planning for circular futures, as fibre blends determine the recycling technologies capable of processing the garment; hard, large, or metal findings need to be removed before recycling to protect equipment; and linings can disrupt the purity of recycled batches [[5], [8]]. Considerations on the composition of waste textile flows are essential for planning which technologies to scale in a circular textile future and determining how waste textiles need to be treated or collected within member states [[1], [5], [8]]. Therefore, detailed data on their presence is critical for planning for a circular transition.

In this sample, only the presence of linings and the number of findings are recorded, as current hand-sorting practices deem a garment ineligible for recycling based on the presence of linings or findings above set thresholds [[2], [3], [5]]. After completing the questionnaire, each item was returned to its place, and the digital survey was submitted for review following the collection period.

After the two collection periods, the surveys were cleaned and compiled into the current dataset. Cleaning consisted of removing incomplete sample surveys from the data set, correcting grammar errors, and ordering fibre composition by highest to lowest percentage. The raw data consisted of 4498 samples; the cleaned data consisted of 4495 samples.

### 4.2 PCTWM Sampling

In the PCTWM dataset, samples were sourced from presorted textile waste from charity and secondhand collection and brought to the SIPTEX sorting plant in Malmo, SE. The sampling was conducted on both pre- and post-NIR sorted bales to ensure the data captured both recyclable and non-recyclable textiles for characterization. Sampling was conducted for three hours per bale by a team of 8 researchers. Each researcher randomly selected a garment from a random position in the broken bale. After sampling the bales were separated by color and each piece was counted. This was done to ensure that a representative sample of the bale was achieved. If there was an underrepresented color group, the appropriate number of items were selected at random from the color group and surveyed.

**Fig. 2:**
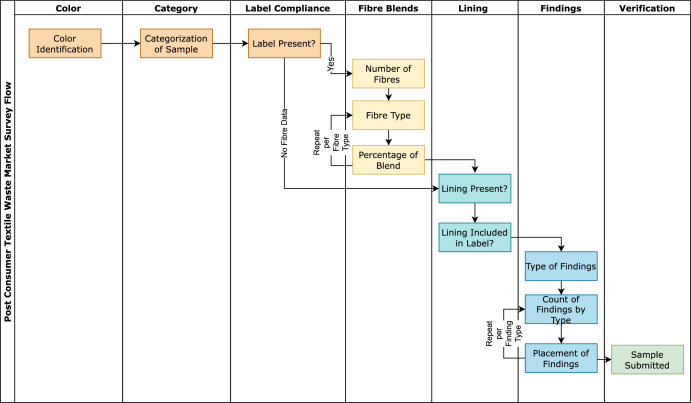
Flow of survey questions to aid in the Post-Consumer Textile Waste Market (PCTWM) field sampling. These questions include a disclosure of the textile category, the presence of a finding declaration and care label, the fibre composition, lining presence, and finding presence.

As in the RMM, garment types follow the definitions in Table 3 and fibre types are standardized as in Table 4. The sampling method for the survey followed the hierarchy illustrated in Fig. 2 and included nine primary criteria: *Color Identification, Textile Categorization, Fibre Declaration and Care Label Presence, Fibre Types, Fibre Composition, Lining Presence and Labeling, Finding Count,* and *Finding Placement*. The garments were first assessed for their color, then their garment category. Researchers then proceeded to locate the fibre label, and record its status as outlined in Table 2. If a label was present and legible, the fibre data was recorded, if not, then “No Fibre Data” was the selected input. Then the researchers identified if the sample contained layers and if the fibre label was inclusive of the label composition, this was recorded following the schema in Table 2. Finally, the findings were classified by material type, counted, and their placement recorded.

**Fig. 3:**
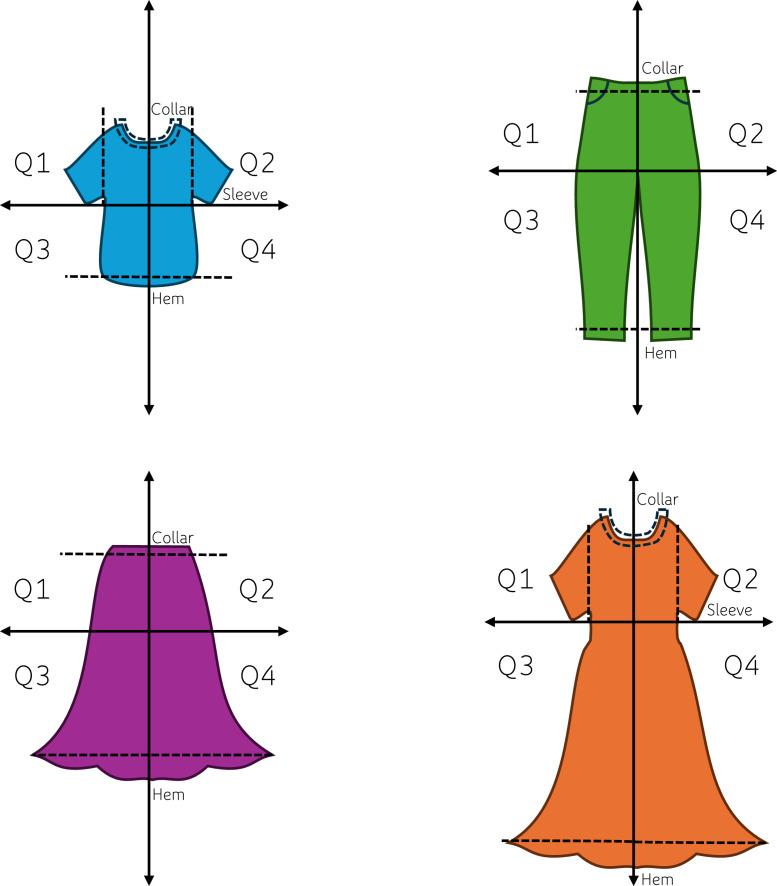
Quadrant method for determining placement of findings on garments in Post-Consumer Textile Waste Market (PCTWM) sampling. Quadrants are further delineated by the front (FQ) or back (BQ) of the garment in the database. Q1 always start from the upper left hand quarter facing the surveyor for both the front and the back when facing the surveyor. Therefore, FQ1 corresponds to BQ2 and so on.

Finding placement was recorded per section of the garment on which the finding type was found. The method for sectioning adopted in this study is illustrated in Fig. 3. In this study, findings can be placed on the front or the back of the garments and may occur on any of the following: *Quadrant 1 (Q1), Quadrant 2 (Q2), Quadrant 3 (Q3), Quadrant 4 (Q4), Sleeves, Collar,* or *Hem.* In the case of tops and sweaters, the quadrants are created by folding the garment in half, so the collar meets the hem and then in half lengthwise, so the sleeves meet. In bottoms, Q1-2 and Q3-4 are separated by folding the top(collar) towards the hem at the bottom of the crotch seam (or at the base of the hip in the case of skirts). In dresses and jackets upper and lower quadrants are separated by folding the collar towards the hem at the top of the midriff (slightly below the end of the bottom of the armhole or sleeve). These sections were determined by identifying the easiest method to quarter the garments if a guillotine cutter were used separating finding heavy quadrants from the garment. Not only is this useful in determining trends in finding placement, but this can be used for identifying where to cut and separate disrupting materials from otherwise recyclable garments.

After the two-day sampling period the surveys were checked, and all incomplete samples were removed. The final data was then cleaned and compiled into the current dataset following the same routine as the RMM dataset (correcting grammar errors from inputs, ordering fibre composition by largest contribution first, and ensuring adherence to adopted nomenclature). The PCTWM raw data contained 1256 samples; the cleaned and available database consists of 1248 samples.

## Limitations

The primary limitations of this data are the geographic location of the sampling, and the seasonality of the garments sampled. The geographic location of DK for the RMM and SE for the PCTWM data is most representative for the Nordic regions. Moreover, the RMM data was collected during the spring and summer collections. Therefore, as garment needs change seasonally and trends change throughout a year, this data may be less representative in certain years and seasons than others. However, these limitations are minor, and as the fibre, findings, and lining types are consistent across both datasets, this database can be used to conduct MFAS and LCAs of common trends in textiles per category.

In addition, the PCTWM sampling was conducted on bales of textiles sort into tops and bottoms and pre-sorted by charity/second-hand actors in the area. That means these bales are assumed to be missing “resaleable” or “reuseable” textiles. Therefore, denim is overrepresented in the bottoms post-sorting bottoms bale samples. As denim has a high number of average disruptors per garment, users should pay attention to the “Article Category Detail” column in the dataset to ensure they take an accurate assessment of the denim sample population.

## Ethics Statement

The authors have read and followed the ethical requirements for publication in Data in Brief. We confirm that the current work does not involve human subjects, animal experiments, or any data from social media platforms.

## Declaration of Generative AI and AI-Assisted Technologies in the Writing Process

During the preparation of this work the author(s) used Co-Pilot in order to review the grammar of the manuscript. After using this tool/service, the author(s) reviewed and edited the content as needed and take(s) full responsibility for the content of the publication.

## CRediT authorship contribution statement

**Heather Margaret Logan:** Conceptualization, Methodology, Writing – original draft, Writing – review & editing, Data curation, Investigation, Investigation, Supervision. **Maggie Ziggie Søndergaard:** Data curation, Investigation, Writing – review & editing. **Valentina Rossi:** Data curation, Investigation, Writing – review & editing. **Kamilla Kastrup Hansen:** Investigation, Writing – review & editing. **Anders Damgaard:** Supervision, Investigation, Writing – review & editing.

## Data Availability

DTU DATANordic Textile Anatomy Database: Composition of Garments Available in the Nordic Retail Mass Market and Post-Consumer Textile Waste Market. (Original data).
